# Tetra­kis(pyridine-κ*N*)bis­(tetrafluorido­borato-κ*F*)copper(II)

**DOI:** 10.1107/S1600536813018643

**Published:** 2013-07-13

**Authors:** Nirosha De Silva, Ajay Pal Singh Pannu, Paul G. Plieger

**Affiliations:** a Institute of Fundamental Sciences, Massey University, Private Bag 11 222, Palmerston North, New Zealand

## Abstract

In the title complex, [Cu(BF_4_)_2_(C_5_H_5_N)_2_], the Cu^II^ ion is in an octa­hedral coordination environment and is surrounded by four pyridine and two tetra­fluoridoborate mol­ecules. The four pyridine mol­ecules are coordinated to the copper ion through their N atoms in the equatorial plane and display a right-handed screw arrangement around the Cu^II^ ion. The remaining two *trans* positions in the octa­hedron are occupied by the BF_4_^−^ anions, each coordinating weakly through an F atom. The crystal packing shows a two-dimensional sheet structure parallel to the *ab* plane that is formed by C—H⋯F hydrogen-bonding inter­actions.

## Related literature

For related [Cu(C_5_H_5_N)_4_ *Y*_2_] complexes (where *Y* = ClO_4_^−^, NO_3_^−^, BF_4_^−^, PF_6_^−^, SO_3_CF_3_^−^) see: Ibers (1953 ▶); Brown *et al.* (1966 ▶); Alleyne & Thompson (1974 ▶); Pradilla Sorzano *et al.* (1979 ▶); Barker & Stobart (1980 ▶); Haynes *et al.* (1988 ▶); Agnus *et al.* (1994 ▶); Beurskens *et al.* (1995 ▶); Li & Zhang (2004 ▶); Bowmaker *et al.* (2011 ▶). For Cu^II^ complexes containing an N_4_F_2_ donor set, see: Su & Li (1994 ▶); Heier *et al.* (1998 ▶); Conner *et al.* (2006 ▶); Noro *et al.* (2009 ▶, 2011 ▶).
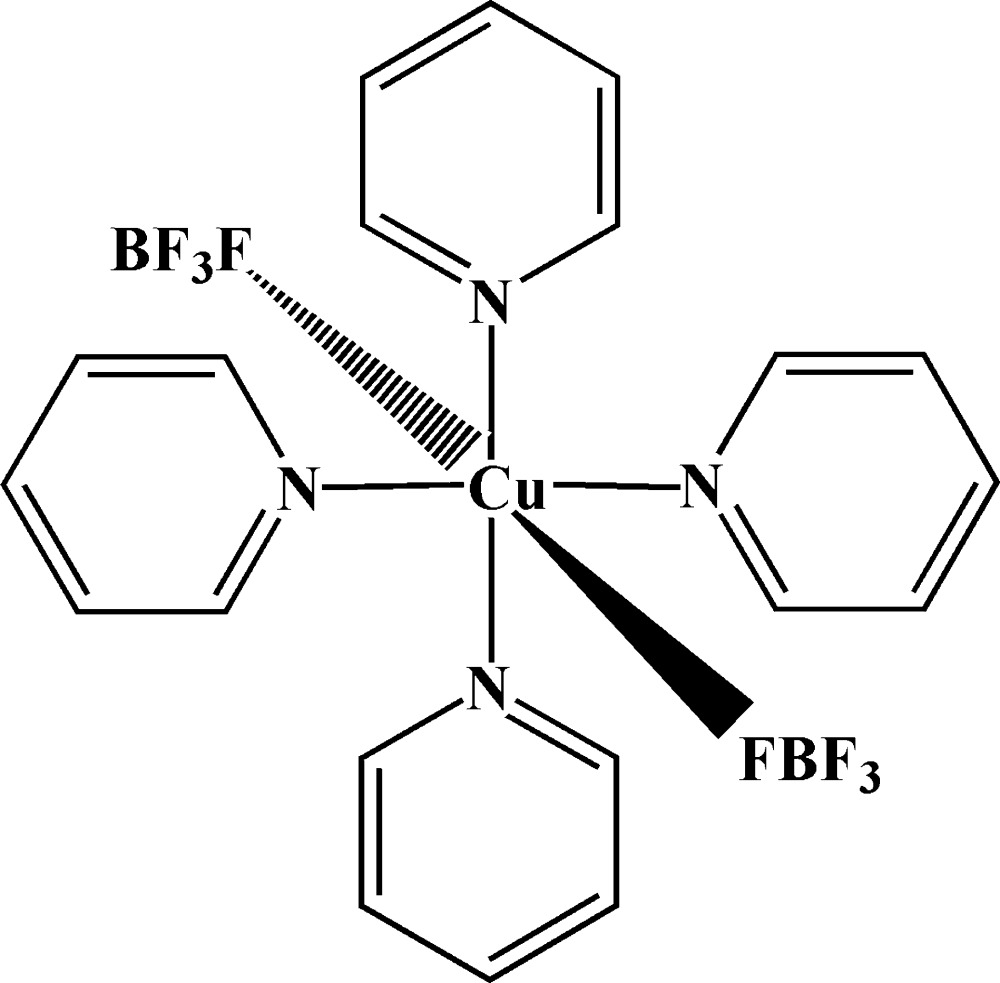


## Experimental

### 

#### Crystal data

[Cu(BF_4_)_2_(C_5_H_5_N)_4_]
*M* *_r_* = 553.56
Orthorhombic, 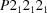

*a* = 10.162 (3) Å
*b* = 13.831 (5) Å
*c* = 16.350 (4) Å
*V* = 2298.0 (12) Å^3^
*Z* = 4
Cu *K*α radiation
μ = 2.10 mm^−1^
*T* = 295 K
0.20 × 0.14 × 0.14 mm

#### Data collection

Rigaku Spider X-ray diffractometer
Absorption correction: multi-scan (*CrystalClear-SM Expert*; Rigaku, 2005 ▶) *T*_min_ = 0.769, *T*_max_ = 1
17937 measured reflections
4378 independent reflections
3186 reflections with *I* > 2σ(*I*)
*R*_int_ = 0.065

#### Refinement

*R*[*F* ^2^ > 2σ(*F* ^2^)] = 0.049
*wR*(*F* ^2^) = 0.117
*S* = 1.01
4378 reflections
317 parameters
H-atom parameters constrained
Δρ_max_ = 0.39 e Å^−3^
Δρ_min_ = −0.68 e Å^−3^
Absolute structure: Flack (1983 ▶), 1868 Friedel pairs
Flack parameter: 0.22 (5)

### 

Data collection: *CrystalClear-SM Expert* (Rigaku, 2005 ▶); cell refinement: *CrystalClear-SM Expert*; data reduction: *CrystalClear-SM Expert*; program(s) used to solve structure: *SIR92* (Altomare *et al.*, 1993 ▶); program(s) used to refine structure: *SHELXL97* (Sheldrick, 2008 ▶); molecular graphics: *ORTEP-3 for Windows* (Farrugia, 2012 ▶); software used to prepare material for publication: *WinGX* (Farrugia, 2012 ▶).

## Supplementary Material

Crystal structure: contains datablock(s) I, New_Global_Publ_Block. DOI: 10.1107/S1600536813018643/sj5342sup1.cif

Structure factors: contains datablock(s) I. DOI: 10.1107/S1600536813018643/sj5342Isup2.hkl

Additional supplementary materials:  crystallographic information; 3D view; checkCIF report

## Figures and Tables

**Table 1:** Hydrogen-bond geometry (Å, °)

*D*—H⋯*A*	*D*—H	H⋯*A*	*D*⋯*A*	*D*—H⋯*A*
C7—H7⋯F5^i^	0.93	2.63	3.016 (6)	105
C7—H7⋯F8^i^	0.93	2.51	3.380 (6)	155
C13—H13⋯F6^ii^	0.93	2.5	3.135 (6)	126
C17—H17⋯F5^iii^	0.93	2.51	3.169 (6)	128

Symmetry codes: (i) 
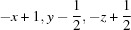
; (ii) 
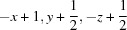
; (iii) 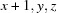.

**Fig. 1.**
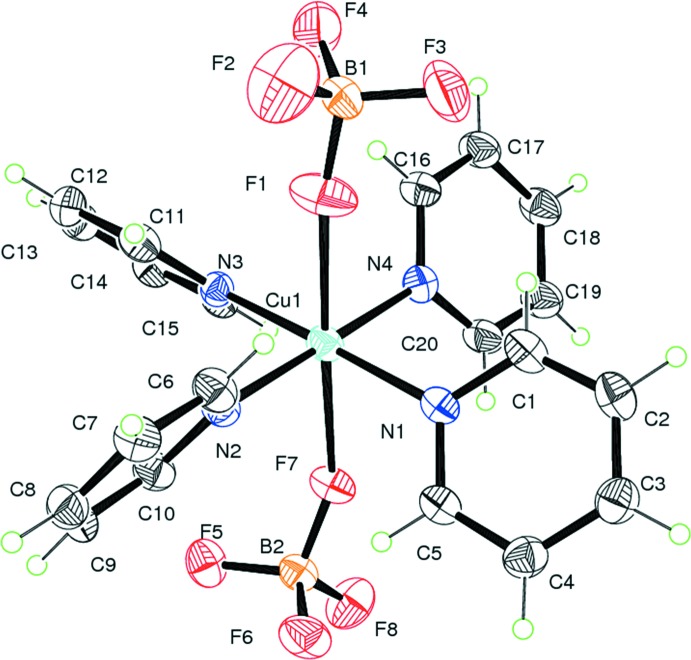
An ORTEP diagram showing the molecular structure of the title complex. The ellipsoids are drawn at 50% probability level. H atoms are presented as a small spheres of arbitrary radius.

**Fig. 2.**
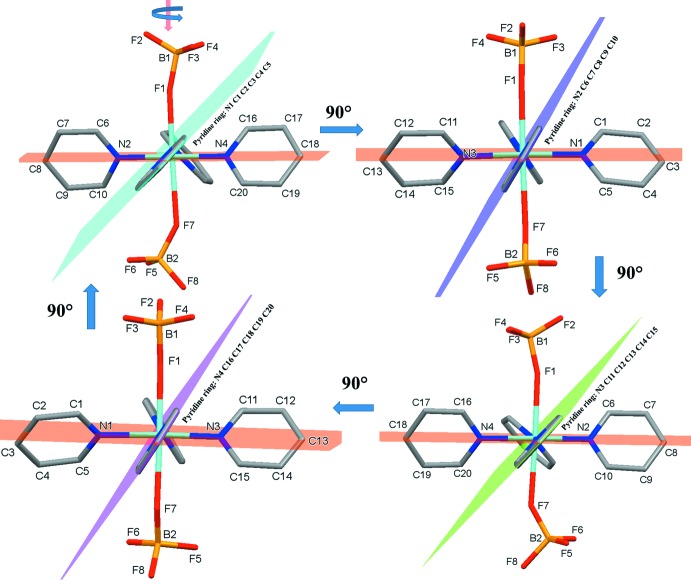
Showing the similarity in orientation and right handed screw arrangement of all the four coordinated pyridine rings with respect to the central metal ion.

**Fig. 3.**
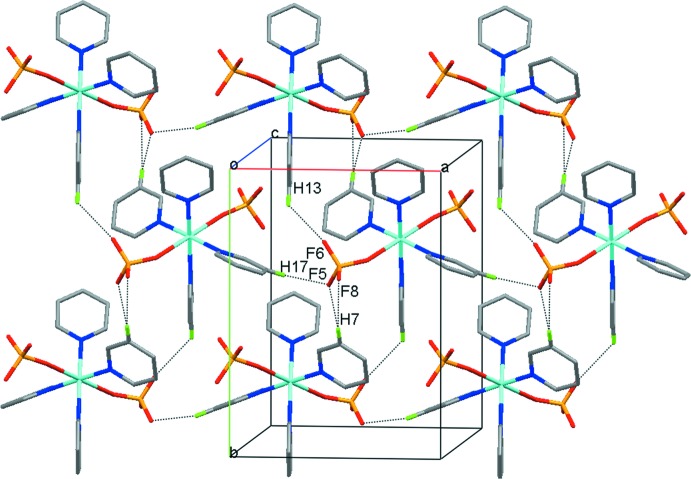
A two-dimensional sheet structure parallel to ab plane is formed by C—H···F hydrogen bonding interactions. The hydrogen atoms other than those involved in H-bonding have been omitted for clarity. Hydrogen bonds are shown in dashed lines.

## supplementary crystallographic information

### Comment

X-ray data on complexes of the type [Cu(C_5_H_5_N)_4_Y_2_] (Y = ClO_4_^-^, NO_3_^-^, BF_4_^-^, PF_6_^-^, NCS^-^, I_3_^-^, SO_3_CF_3_^-^, CF_3_CO_2_^-^) started appearing from the 1950's onwards (Ibers, 1953; Brown *et al.*, 1966; Alleyne *et al.*, 1974; Pradilla *et al.*, 1979; Barker *et al.*, 1980; Haynes *et al.*, 1988; Agnus *et al.*, 1994; Beurskens *et al.*, 1995; Li *et al.*, 2004; Bowmaker *et al.*, 2011). Among these complexes, preliminary structural investigations on [Cu(C_5_H_5_N)_2_(BF_4_)_2_] were carried out by Ibers (Ibers, 1953) but were limited to the unit cell and space group determination. At that time because of the size and complexity of the unit cell no further work was completed. Based on Ibers' analysis, Agnus and co-workers in their paper on the structural determination of the related complex [Cu(C_5_H_5_N)_4_(ClO_4_)_2_], predicted that the tetrafluoridoborate analogue would also crystalize as a structural enantiomer. A CCDC search reveals that although there have been many reports on Cu^II^ complexes with the [Cu(C_5_H_5_N)_4_Y_2_] structural motif there are no single-crystal X-ray reports with BF_4_ as the counter ion (Y = BF_4_). Therefore, in the present work we report the single-crystal X-ray analysis of this complex, [Cu(C_5_H_5_N)_2_(BF_4_)_2_].

The molecular structure of present complex is shown in Fig. 1 along with the atom labelling scheme. In this complex, the four pyridine ligand molecules are coordinating through their nitrogen atoms forming a square plane around the Cu^II^ center while the remaining two *trans* positions in the distorted octahedron are occupied by the BF_4_ molecules, each coordinating through an F atom. The Cu—N distance varies from 2.009 (4) Å to 2.037 (4) Å while the two long Cu—F *trans* distances of 2.406 (4) Å and 2.476 (3) Å are consistent with other hexacoordinate Cu^II^ complexes containing an N_4_F_2_ donor set (2.394 (3) Å, Su *et al.*, 1994; 2.452 (3) Å, Heier *et al.*, 1998; 2.376 (2) Å, Conner *et al.*, 2006; 2.501 (3) – 2.503 (3) Å, Noro *et al.*, 2011; 2.528 (3) – 2.587 (2) Å, Noro *et al.*, 2009). The dihedral angle values of 47.6 (3)° (N1—Cu1—N4—C25), 58.4 (3)° (N2—Cu1—N1—C6), 40.7 (2)° (N3—Cu1—N2—C1) and 58.3 (3)° (N4—Cu1—N3—C20) indicate a similar orientation for each pyridine ring with respect to the equatorial plane (plane containing the Cu^II^ and the four coordinated nitrogen atoms). Fig. 2. gives a pictorial view of this similarity in orientation along with confirming the right handed screw arrangement of all the four coordinated pyridine rings. This as predicted by Agnus and co-workers is very similar to the isomorphous perchlorate complex [Cu(C_5_H_5_N)_4_(ClO_4_)_2_] (Agnus *et al.*, 1994) which also crystalizes in orthorhombic crystal system with *P*2_1_2_1_2_1_ space group and similar unit-cell parameters. The crystal packing investigations in present complex show a two-dimensional sheet structure parallel to the *ab* plane is formed by C—H···F hydrogen bonding interactions (Fig. 3, Table 1).

### Experimental

Crystals of [Cu(C_5_H_5_N)_4_(BF_4_)_2_] were obtained by the slow evaporation of a mixed solvent solution (MeOH: H_2_O: pyridine, 15: 10: 5 ml respectively) containing copper(II) tetrafluoridoborate hexahydrate (0.345 g, 1.0 mmol). Blue crystals of [Cu(C_5_H_5_N)_2_(BF_4_)_2_] were obtained after 2–3 weeks from the filtrate.

### Refinement

All non-hydrogen atoms were refined anisotropically. All H atoms were positioned geometrically with C–H = 0.93 and constrained to ride on their parent atoms, with Uiso(H) = 1.2Ueq(C). The crystal studied was an inversion twin with a 0.78 (7):0.22 (7) domain ratio.

### Crystal data

**Table:**

[Cu(BF_4_)_2_(C_5_H_5_N)_4_]	*F*(000) = 1116
*M**_r_* = 553.56	*D*_x_ = 1.6 Mg m^−^^3^
Orthorhombic, *P*2_1_2_1_2_1_	Cu *K*α radiation, λ = 1.54178 Å
Hall symbol: P 2ac 2ab	Cell parameters from 2453 reflections
*a* = 10.162 (3) Å	θ = 7–71.4°
*b* = 13.831 (5) Å	µ = 2.10 mm^−^^1^
*c* = 16.350 (4) Å	*T* = 295 K
*V* = 2298.0 (12) Å^3^	Block, blue
*Z* = 4	0.2 × 0.14 × 0.14 mm

### Data collection

**Table:**

Rigaku Spider X-ray diffractometer	4378 independent reflections
Radiation source: Rotating Anode	3186 reflections with *I* > 2σ(*I*)
Graphite monochromator	*R*_int_ = 0.065
Detector resolution: 10 pixels mm^-1^	θ_max_ = 71.8°, θ_min_ = 7.0°
profile data from ω–scans	*h* = −10→12
Absorption correction: multi-scan (*CrystalClear-SM Expert*; Rigaku, 2005)	*k* = −16→16
*T*_min_ = 0.769, *T*_max_ = 1	*l* = −19→20
17937 measured reflections	

### Refinement

**Table:**

Refinement on *F*^2^	Secondary atom site location: difference Fourier map
Least-squares matrix: full	Hydrogen site location: inferred from neighbouring sites
*R*[*F*^2^ > 2σ(*F*^2^)] = 0.049	H-atom parameters constrained
*wR*(*F*^2^) = 0.117	*w* = 1/[σ^2^(*F*_o_^2^) + (0.030*P*)^2^ + 2.2291*P*] where *P* = (*F*_o_^2^ + 2*F*_c_^2^)/3
*S* = 1.01	(Δ/σ)_max_ < 0.001
4378 reflections	Δρ_max_ = 0.39 e Å^−^^3^
317 parameters	Δρ_min_ = −0.68 e Å^−^^3^
0 restraints	Absolute structure: Flack (1983), 1868 Friedel pairs
Primary atom site location: structure-invariant direct methods	Flack parameter: 0.22 (5)

### Special details

**Table:**

Geometry. All e.s.d.'s (except the e.s.d. in the dihedral angle between two l.s. planes) are estimated using the full covariance matrix. The cell e.s.d.'s are taken into account individually in the estimation of e.s.d.'s in distances, angles and torsion angles; correlations between e.s.d.'s in cell parameters are only used when they are defined by crystal symmetry. An approximate (isotropic) treatment of cell e.s.d.'s is used for estimating e.s.d.'s involving l.s. planes.
Refinement. Refinement of *F*^2^ against ALL reflections. The weighted *R*-factor *wR* and goodness of fit *S* are based on *F*^2^, conventional *R*-factors *R* are based on *F*, with *F* set to zero for negative *F*^2^. The threshold expression of *F*^2^ > σ(*F*^2^) is used only for calculating *R*-factors(gt) *etc*. and is not relevant to the choice of reflections for refinement. *R*-factors based on *F*^2^ are statistically about twice as large as those based on *F*, and *R*- factors based on ALL data will be even larger.

### Fractional atomic coordinates and isotropic or equivalent isotropic displacement parameters (Å^2^)

**Table:**

	*x*	*y*	*z*	*U*_iso_*/*U*_eq_	
C1	0.8793 (5)	0.0901 (4)	0.1770 (3)	0.0447 (12)	
H1	0.9558	0.1139	0.2008	0.054*	
C2	0.8868 (5)	0.0101 (3)	0.1268 (3)	0.0484 (13)	
H2	0.9676	−0.0192	0.1168	0.058*	
C3	0.7746 (5)	−0.0259 (3)	0.0918 (3)	0.0473 (12)	
H3	0.7787	−0.0789	0.0568	0.057*	
C4	0.6545 (5)	0.0176 (4)	0.1092 (3)	0.0474 (13)	
H4	0.5764	−0.0068	0.088	0.057*	
C5	0.6548 (5)	0.0982 (4)	0.1591 (3)	0.0424 (12)	
H5	0.575	0.1285	0.17	0.051*	
C6	0.6032 (5)	0.1434 (3)	0.3647 (3)	0.0449 (12)	
H6	0.6802	0.1072	0.368	0.054*	
C7	0.4944 (5)	0.1140 (4)	0.4088 (3)	0.0454 (12)	
H7	0.4982	0.0594	0.4418	0.054*	
C8	0.3803 (5)	0.1671 (4)	0.4029 (3)	0.0465 (12)	
H8	0.3057	0.1489	0.4322	0.056*	
C9	0.3779 (4)	0.2478 (3)	0.3530 (3)	0.0428 (11)	
H9	0.3012	0.2838	0.3473	0.051*	
C10	0.4888 (4)	0.2738 (3)	0.3124 (3)	0.0422 (11)	
H10	0.4871	0.3291	0.28	0.051*	
C20	0.9163 (5)	0.3097 (4)	0.1054 (3)	0.0471 (13)	
H20	0.8419	0.2878	0.078	0.057*	
C19	1.0217 (5)	0.3422 (4)	0.0604 (3)	0.0523 (14)	
H19	1.0172	0.3436	0.0036	0.063*	
C18	1.1332 (5)	0.3726 (4)	0.0996 (3)	0.0500 (13)	
H18	1.2053	0.3945	0.0698	0.06*	
C17	1.1373 (5)	0.3703 (4)	0.1849 (3)	0.0500 (13)	
H17	1.2121	0.3897	0.2132	0.06*	
C16	1.0275 (5)	0.3384 (3)	0.2257 (3)	0.0456 (12)	
H16	1.0294	0.3375	0.2826	0.055*	
C15	0.7589 (4)	0.4783 (3)	0.2670 (3)	0.0424 (10)	
H15	0.7678	0.4753	0.2104	0.051*	
C14	0.7524 (5)	0.5676 (3)	0.3032 (3)	0.0470 (11)	
H14	0.7554	0.6236	0.2718	0.056*	
C13	0.7415 (5)	0.5726 (3)	0.3870 (3)	0.0503 (12)	
H13	0.7395	0.6324	0.413	0.06*	
C12	0.7335 (5)	0.4887 (3)	0.4318 (3)	0.0487 (12)	
H12	0.7245	0.4908	0.4884	0.058*	
C11	0.7390 (5)	0.4010 (3)	0.3908 (3)	0.0431 (11)	
H11	0.7329	0.3442	0.421	0.052*	
B1	1.0010 (6)	0.1685 (5)	0.4073 (4)	0.0490 (15)	
B2	0.4990 (6)	0.3467 (4)	0.1079 (4)	0.0454 (14)	
N2	0.6013 (4)	0.2225 (3)	0.3173 (2)	0.0385 (9)	
N1	0.7637 (4)	0.1349 (3)	0.1924 (2)	0.0381 (8)	
N4	0.9169 (4)	0.3084 (3)	0.1878 (2)	0.0397 (9)	
N3	0.7529 (4)	0.3950 (2)	0.30907 (19)	0.0341 (8)	
F1	0.8975 (3)	0.1997 (3)	0.3593 (2)	0.0807 (11)	
F2	0.9527 (5)	0.1251 (3)	0.4732 (2)	0.1131 (15)	
F3	1.0755 (3)	0.1081 (3)	0.3611 (3)	0.1038 (14)	
F4	1.0738 (3)	0.2493 (3)	0.4275 (2)	0.0841 (11)	
F5	0.4395 (3)	0.41614 (19)	0.15695 (18)	0.0565 (8)	
F6	0.4263 (3)	0.2628 (2)	0.1097 (2)	0.0658 (9)	
F7	0.6239 (3)	0.3276 (2)	0.13865 (18)	0.0545 (7)	
F8	0.5108 (4)	0.3811 (2)	0.02866 (18)	0.0716 (10)	
Cu1	0.75983 (6)	0.26389 (4)	0.25232 (4)	0.03806 (18)	

### Atomic displacement parameters (Å^2^)

**Table:**

	*U*^11^	*U*^22^	*U*^33^	*U*^12^	*U*^13^	*U*^23^
C1	0.037 (3)	0.040 (3)	0.056 (3)	0.001 (2)	0.005 (2)	0.009 (2)
C2	0.043 (3)	0.041 (3)	0.062 (3)	0.006 (2)	0.010 (3)	0.009 (3)
C3	0.055 (3)	0.040 (3)	0.047 (3)	−0.001 (3)	0.004 (2)	−0.002 (2)
C4	0.049 (3)	0.042 (3)	0.052 (3)	−0.003 (3)	−0.003 (2)	−0.004 (2)
C5	0.034 (3)	0.042 (3)	0.051 (3)	0.001 (2)	−0.002 (2)	0.002 (2)
C6	0.041 (3)	0.039 (3)	0.054 (3)	0.002 (2)	0.000 (2)	−0.001 (2)
C7	0.049 (3)	0.040 (3)	0.048 (3)	−0.005 (3)	0.003 (2)	0.003 (2)
C8	0.043 (3)	0.052 (3)	0.045 (3)	−0.006 (3)	0.008 (2)	−0.006 (2)
C9	0.034 (2)	0.047 (3)	0.048 (2)	0.005 (2)	0.004 (2)	−0.002 (2)
C10	0.040 (3)	0.039 (3)	0.047 (2)	0.003 (2)	−0.003 (2)	0.003 (2)
C20	0.033 (3)	0.052 (3)	0.057 (3)	−0.006 (2)	−0.003 (2)	−0.002 (3)
C19	0.042 (3)	0.066 (4)	0.048 (3)	−0.006 (3)	0.002 (2)	0.007 (3)
C18	0.038 (3)	0.064 (4)	0.048 (3)	−0.003 (3)	0.007 (2)	0.007 (3)
C17	0.034 (3)	0.063 (3)	0.053 (3)	−0.008 (3)	0.001 (2)	0.003 (3)
C16	0.037 (3)	0.050 (3)	0.050 (3)	−0.005 (2)	−0.001 (2)	0.001 (2)
C15	0.035 (3)	0.042 (2)	0.051 (3)	0.000 (2)	0.003 (2)	0.004 (2)
C14	0.045 (3)	0.041 (3)	0.054 (3)	0.005 (3)	0.001 (3)	0.009 (2)
C13	0.044 (3)	0.039 (3)	0.068 (3)	0.005 (3)	0.000 (3)	−0.002 (2)
C12	0.047 (3)	0.051 (3)	0.049 (3)	0.004 (3)	−0.001 (3)	−0.007 (2)
C11	0.038 (3)	0.043 (2)	0.048 (2)	0.001 (3)	0.000 (2)	0.009 (2)
B1	0.037 (4)	0.056 (4)	0.055 (3)	0.003 (3)	0.006 (3)	0.000 (3)
B2	0.035 (3)	0.044 (3)	0.057 (3)	−0.001 (3)	0.000 (3)	0.005 (3)
N2	0.035 (2)	0.036 (2)	0.0443 (19)	−0.0009 (18)	0.0007 (17)	−0.0012 (17)
N1	0.030 (2)	0.038 (2)	0.0459 (19)	0.003 (2)	−0.0002 (18)	0.0019 (16)
N4	0.037 (2)	0.041 (2)	0.041 (2)	−0.0015 (19)	0.0015 (18)	−0.0003 (18)
N3	0.034 (2)	0.0341 (18)	0.0344 (16)	−0.0019 (19)	0.0024 (18)	0.0004 (14)
F1	0.055 (2)	0.092 (3)	0.095 (2)	0.0015 (19)	−0.0267 (19)	0.030 (2)
F2	0.164 (4)	0.104 (3)	0.071 (2)	−0.012 (3)	0.017 (3)	0.035 (2)
F3	0.054 (2)	0.107 (3)	0.149 (4)	0.014 (2)	0.011 (2)	−0.063 (3)
F4	0.060 (2)	0.094 (3)	0.098 (2)	−0.024 (2)	0.0140 (18)	−0.044 (2)
F5	0.0586 (19)	0.0407 (16)	0.0703 (19)	0.0070 (14)	0.0111 (16)	0.0029 (15)
F6	0.0495 (18)	0.0472 (18)	0.101 (2)	−0.0135 (15)	0.0000 (16)	−0.0033 (18)
F7	0.0362 (16)	0.0633 (19)	0.0641 (18)	0.0026 (14)	−0.0068 (14)	0.0026 (15)
F8	0.103 (3)	0.064 (2)	0.0479 (16)	0.007 (2)	−0.0089 (18)	0.0095 (15)
Cu1	0.0327 (3)	0.0364 (3)	0.0450 (3)	−0.0019 (3)	0.0014 (3)	0.0001 (3)

### Geometric parameters (Å, º)

**Table:**

C1—N1	1.352 (6)	C17—C16	1.373 (6)
C1—C2	1.380 (7)	C17—H17	0.93
C1—H1	0.93	C16—N4	1.349 (5)
C2—C3	1.370 (7)	C16—H16	0.93
C2—H2	0.93	C15—N3	1.344 (5)
C3—C4	1.391 (7)	C15—C14	1.371 (6)
C3—H3	0.93	C15—H15	0.93
C4—C5	1.381 (7)	C14—C13	1.376 (6)
C4—H4	0.93	C14—H14	0.93
C5—N1	1.333 (6)	C13—C12	1.375 (6)
C5—H5	0.93	C13—H13	0.93
C6—N2	1.341 (6)	C12—C11	1.387 (6)
C6—C7	1.381 (6)	C12—H12	0.93
C6—H6	0.93	C11—N3	1.346 (5)
C7—C8	1.376 (7)	C11—H11	0.93
C7—H7	0.93	B1—F2	1.328 (7)
C8—C9	1.383 (7)	B1—F3	1.357 (7)
C8—H8	0.93	B1—F4	1.381 (7)
C9—C10	1.356 (6)	B1—F1	1.382 (6)
C9—H9	0.93	B2—F6	1.375 (6)
C10—N2	1.348 (5)	B2—F5	1.390 (6)
C10—H10	0.93	B2—F7	1.390 (6)
C20—N4	1.347 (6)	B2—F8	1.386 (6)
C20—C19	1.375 (7)	N2—Cu1	2.013 (4)
C20—H20	0.93	N1—Cu1	2.036 (4)
C19—C18	1.368 (7)	N4—Cu1	2.010 (4)
C19—H19	0.93	N3—Cu1	2.038 (3)
C18—C17	1.396 (6)	F1—Cu1	2.409 (3)
C18—H18	0.93		
			
N1—C1—C2	121.7 (5)	C13—C14—C15	118.7 (4)
N1—C1—H1	119.1	C13—C14—H14	120.7
C2—C1—H1	119.1	C15—C14—H14	120.7
C3—C2—C1	119.6 (5)	C14—C13—C12	119.5 (4)
C3—C2—H2	120.2	C14—C13—H13	120.2
C1—C2—H2	120.2	C12—C13—H13	120.2
C2—C3—C4	119.2 (4)	C13—C12—C11	118.5 (4)
C2—C3—H3	120.4	C13—C12—H12	120.7
C4—C3—H3	120.4	C11—C12—H12	120.7
C5—C4—C3	118.0 (5)	N3—C11—C12	122.6 (4)
C5—C4—H4	121	N3—C11—H11	118.7
C3—C4—H4	121	C12—C11—H11	118.7
N1—C5—C4	123.4 (5)	F2—B1—F3	112.3 (6)
N1—C5—H5	118.3	F2—B1—F4	111.7 (5)
C4—C5—H5	118.3	F3—B1—F4	109.5 (5)
N2—C6—C7	122.0 (5)	F2—B1—F1	108.7 (5)
N2—C6—H6	119	F3—B1—F1	107.5 (5)
C7—C6—H6	119	F4—B1—F1	107.0 (5)
C8—C7—C6	118.8 (5)	F6—B2—F5	109.7 (4)
C8—C7—H7	120.6	F6—B2—F7	108.8 (4)
C6—C7—H7	120.6	F5—B2—F7	108.7 (4)
C9—C8—C7	119.1 (5)	F6—B2—F8	110.9 (5)
C9—C8—H8	120.5	F5—B2—F8	109.8 (4)
C7—C8—H8	120.5	F7—B2—F8	108.9 (5)
C10—C9—C8	119.3 (4)	C6—N2—C10	118.4 (4)
C10—C9—H9	120.4	C6—N2—Cu1	121.7 (3)
C8—C9—H9	120.4	C10—N2—Cu1	119.9 (3)
N2—C10—C9	122.4 (4)	C5—N1—C1	118.1 (4)
N2—C10—H10	118.8	C5—N1—Cu1	120.9 (3)
C9—C10—H10	118.8	C1—N1—Cu1	120.5 (3)
N4—C20—C19	122.4 (5)	C20—N4—C16	117.3 (4)
N4—C20—H20	118.8	C20—N4—Cu1	121.7 (3)
C19—C20—H20	118.8	C16—N4—Cu1	121.0 (3)
C18—C19—C20	119.6 (5)	C15—N3—C11	117.3 (4)
C18—C19—H19	120.2	C15—N3—Cu1	121.9 (3)
C20—C19—H19	120.2	C11—N3—Cu1	120.7 (3)
C19—C18—C17	119.1 (5)	B1—F1—Cu1	165.8 (3)
C19—C18—H18	120.5	N4—Cu1—N2	178.68 (15)
C17—C18—H18	120.5	N4—Cu1—N3	89.65 (15)
C16—C17—C18	117.9 (5)	N2—Cu1—N3	89.15 (14)
C16—C17—H17	121	N4—Cu1—N1	90.04 (15)
C18—C17—H17	121	N2—Cu1—N1	91.15 (15)
N4—C16—C17	123.6 (4)	N3—Cu1—N1	178.11 (14)
N4—C16—H16	118.2	N4—Cu1—F1	91.87 (14)
C17—C16—H16	118.2	N2—Cu1—F1	88.69 (14)
N3—C15—C14	123.3 (4)	N3—Cu1—F1	91.01 (13)
N3—C15—H15	118.3	N1—Cu1—F1	90.87 (14)
C14—C15—H15	118.3		
			
N1—C1—C2—C3	−0.4 (7)	F2—B1—F1—Cu1	−173.6 (12)
C1—C2—C3—C4	−1.6 (7)	F3—B1—F1—Cu1	−51.8 (17)
C2—C3—C4—C5	2.4 (7)	F4—B1—F1—Cu1	65.6 (16)
C3—C4—C5—N1	−1.4 (7)	C20—N4—Cu1—N3	−121.7 (4)
N2—C6—C7—C8	0.8 (7)	C16—N4—Cu1—N3	58.2 (4)
C6—C7—C8—C9	0.2 (7)	C20—N4—Cu1—N1	56.5 (4)
C7—C8—C9—C10	−1.4 (7)	C16—N4—Cu1—N1	−123.6 (4)
C8—C9—C10—N2	1.6 (7)	C20—N4—Cu1—F1	147.3 (4)
N4—C20—C19—C18	1.6 (8)	C16—N4—Cu1—F1	−32.8 (4)
C20—C19—C18—C17	−0.3 (8)	C6—N2—Cu1—N3	−123.6 (4)
C19—C18—C17—C16	−0.8 (8)	C10—N2—Cu1—N3	57.7 (3)
C18—C17—C16—N4	0.7 (8)	C6—N2—Cu1—N1	58.3 (4)
N3—C15—C14—C13	1.1 (8)	C10—N2—Cu1—N1	−120.4 (3)
C15—C14—C13—C12	−1.9 (9)	C6—N2—Cu1—F1	−32.6 (4)
C14—C13—C12—C11	1.1 (8)	C10—N2—Cu1—F1	148.7 (3)
C13—C12—C11—N3	0.5 (8)	C15—N3—Cu1—N4	48.4 (4)
C7—C6—N2—C10	−0.6 (7)	C11—N3—Cu1—N4	−133.2 (4)
C7—C6—N2—Cu1	−179.3 (4)	C15—N3—Cu1—N2	−131.1 (4)
C9—C10—N2—C6	−0.6 (7)	C11—N3—Cu1—N2	47.4 (4)
C9—C10—N2—Cu1	178.1 (3)	C15—N3—Cu1—F1	140.2 (4)
C4—C5—N1—C1	−0.6 (7)	C11—N3—Cu1—F1	−41.3 (4)
C4—C5—N1—Cu1	171.6 (4)	C5—N1—Cu1—N4	−130.8 (4)
C2—C1—N1—C5	1.5 (7)	C1—N1—Cu1—N4	41.2 (4)
C2—C1—N1—Cu1	−170.7 (3)	C5—N1—Cu1—N2	48.6 (4)
C19—C20—N4—C16	−1.6 (8)	C1—N1—Cu1—N2	−139.4 (3)
C19—C20—N4—Cu1	178.3 (4)	C5—N1—Cu1—F1	137.3 (3)
C17—C16—N4—C20	0.4 (7)	C1—N1—Cu1—F1	−50.7 (3)
C17—C16—N4—Cu1	−179.5 (4)	B1—F1—Cu1—N4	−6.8 (15)
C14—C15—N3—C11	0.5 (7)	B1—F1—Cu1—N2	174.4 (15)
C14—C15—N3—Cu1	179.0 (4)	B1—F1—Cu1—N3	−96.5 (15)
C12—C11—N3—C15	−1.3 (8)	B1—F1—Cu1—N1	83.2 (15)
C12—C11—N3—Cu1	−179.8 (4)		

### Hydrogen-bond geometry (Å, º)

**Table:**

*D*—H···*A*	*D*—H	H···*A*	*D*···*A*	*D*—H···*A*
C7—H7···F5^i^	0.93	2.63	3.016 (6)	105
C7—H7···F8^i^	0.93	2.51	3.380 (6)	155
C13—H13···F6^ii^	0.93	2.5	3.135 (6)	126
C17—H17···F5^iii^	0.93	2.51	3.169 (6)	128

Symmetry codes: (i) −*x*+1, *y*−1/2, −*z*+1/2; (ii) −*x*+1, *y*+1/2, −*z*+1/2; (iii) *x*+1, *y*, *z*.
